# Machine learning in medicine: a practical introduction to techniques for data pre-processing, hyperparameter tuning, and model comparison

**DOI:** 10.1186/s12874-022-01758-8

**Published:** 2022-11-01

**Authors:** André Pfob, Sheng-Chieh Lu, Chris Sidey-Gibbons

**Affiliations:** 1 Department of Obstetrics and Gynecology, University Breast Unit, Heidelberg University Hospital, Heidelberg, Germany; 2 MD Anderson Center for INSPiRED Cancer Care, The University of Texas MD Anderson Cancer Center, Houston, USA; 3 Section of Patient-Centered Analytics, The University of Texas MD Anderson Cancer Center, Houston, TX 77030 USA

**Keywords:** Machine learning, Artificial intelligence, Guideline, Medicine

## Abstract

**Background:**

There is growing enthusiasm for the application of machine learning (ML) and artificial intelligence (AI) techniques to clinical research and practice. However, instructions on how to develop robust high-quality ML and AI in medicine are scarce. In this paper, we provide a practical example of techniques that facilitate the development of high-quality ML systems including data pre-processing, hyperparameter tuning, and model comparison using open-source software and data.

**Methods:**

We used open-source software and a publicly available dataset to train and validate multiple ML models to classify breast masses into benign or malignant using mammography image features and patient age. We compared algorithm predictions to the ground truth of histopathologic evaluation. We provide step-by-step instructions with accompanying code lines.

**Findings:**

Performance of the five algorithms at classifying breast masses as benign or malignant based on mammography image features and patient age was statistically equivalent (*P* > 0.05). Area under the receiver operating characteristics curve (AUROC) for the logistic regression with elastic net penalty was 0.89 (95% CI 0.85 – 0.94), for the Extreme Gradient Boosting Tree 0.88 (95% CI 0.83 – 0.93), for the Multivariate Adaptive Regression Spline algorithm 0.88 (95% CI 0.83 – 0.93), for the Support Vector Machine 0.89 (95% CI 0.84 – 0.93), and for the neural network 0.89 (95% CI 0.84 – 0.93).

**Interpretation:**

Our paper allows clinicians and medical researchers who are interested in using ML algorithms to understand and recreate the elements of a comprehensive ML analysis. Following our instructions may help to improve model generalizability and reproducibility in medical ML studies.

**Supplementary Information:**

The online version contains supplementary material available at 10.1186/s12874-022-01758-8.

## Introduction

Interest in artificial intelligence (AI) and machine learning (ML) has drastically increased over the past years within the global medical community. The use of AI/ML techniques could improve care for patients by providing individualized outcome predictions and by reducing redundancy in standardized processes allowing clinicians to spend more time with patients [1–7]. While protocol and reporting guidelines for clinical trials using AI/ML techniques have recently been published (CONSORT-AI [8] and SPIRIT-AI [9]), high-quality instructions on how to practically develop ML models within the medical context are not abundant. Unfortunately, despite high interest in this area, there are many examples of medical AI that have not been rigorously developed which makes best-practice case studies on how to practically develop these models of high interest for clinical research and practice [10, 11].

Our group has previously published an introductory paper on ML in medicine that explains the general concept of ML and gives a practical introduction to building an ML algorithm using open-source R statistical programming software and open-source data [12]. In the present manuscript, we build on this introduction to explain some more techniques including data pre-processing, hyperparameter tuning, and model comparison with examples using open-source software and data. These steps are essential to improve not only the performance of ML algorithms but also to ensure better generalizability and to provide a balanced evaluation of these algorithms. For interested readers, there is another paper within this series covering the use of natural language in medical AI studies [13].

In brief, data pre-processing consists of two main categories: data cleaning and feature engineering. Data cleaning is the process of removing duplicative, incorrect, and irrelevant data and of addressing missing data, which requires substantial knowledge about the data, the context within which it was collected, and context where the model will be used. Thus, multidisciplinary collaboration between clinicians and data scientists is required to adequately clean the data. Feature engineering uses various statistical approaches to prepare the data that ML algorithms can then better utilize. Common feature engineering procedures include data normalization, transformation, feature selection, dimensionality reduction, and data type conversion to meet the prerequisites of ML algorithms [14].

Machine learning algorithms all have so-called hyperparameters that control the configuration of a specific algorithm. Hyperparameters can be classified into optimization hyperparameters, which generally control the overall training process (e.g. the learning rate), and model hyperparameters, which specify the specific algorithm architecture (e.g., the number of layers in a neural network). In contrast to model parameters, which are directly derived from data during the training process, hyperparameters are manually pre-specified and can usually vary across different models. Hyperparameters are key to model performance for a given task on a specific dataset. The process of identifying the optimal combination of hyperparameters, so-called model tuning or optimization, often makes ML algorithms computationally expensive. The tuned model is then evaluated on a validation dataset that is independent of the training dataset [15].

Finally, we often want to know which model performs best. We conduct statistical tests to compare different models against each other, allowing us to evaluate whether differences in model performance are actually statistically significant. There may be situations in which we do not necessarily wish to deploy the algorithm with the best performance on the testing dataset – to improve generalizability and enable easy implementation. For instance, we may choose the simplest model that is with a certain degree of performance from the best performing model. We may also prioritize other aspects including whether the model output is readily interpretable.

### What this paper will achieve

In this paper, we will provide a practical example of best-practice ML techniques like data pre-processing, hyperparameter tuning, and model comparison using open-source software and data. Our paper is aimed primarily at medical researchers and practitioners who are interested in using and developing ML algorithms for their analyses and who are looking for guidance on how to perform a comprehensive ML analysis on their own.

### How to follow this paper

This paper provides step-by-step instructions on how to perform an ML analysis, starting with the data preparation and ending with the model evaluation. We provide exemplary code using the open-source R statistical programming language throughout this paper (the complete code is available in the Supplementary Appendix). We advise readers who are not familiar with the R programming language to read our introductory paper first that provides guidance on how to use and set up R [12]. We used open source data for this analysis which is freely available at the UCI Machine Learning Repository (“Mammographic Mass” dataset) [16].

## Material and methods

### Dataset

We used open-source data that is freely available at the UCI Machine Learning Repository [16]. The “Mammographic Mass” dataset contains anonymized data from 961 patients who underwent mammography to evaluate an unclear breast lesion. Mammography image features (shape of lesion, margin of lesion, density), patient age, and the results of the histopathologic evaluation (gold standard, benign or malignant) are provided. Of the 961 patients, 516 (53.7%) had a benign breast lesion, and 445 (46.3%) had a malignant breast lesion. This dataset can be used for developing models to predict whether a breast lesion is histopathologically benign or malignant based on the image features and patient age [17].

### Software

R version 4.0.3 was used for all analyses.

### Machine learning analysis

#### Data preparation

We first load the dataset from the UCI repository and label the columns as specified in the dataset description (see Table 1, Task 1.1 and 1.2, for R code) [16]. The resultant dataset is called “db” and has six columns (“BI-RADS”, “Age”, “Shape”, “Margin”, “Density”, and “outcome”).

**Table 1:** Summary of key Tasks and accompanying R code for a machine learning analysis

Task	Code in R language
1) Data preparation	
1.1) Import new dataset called „db “	db <—read.csv (url ("https://archive.ics.uci.edu/ml/machine-learning-databases/mammographic-masses/mammographic_masses.data"))
1.2) Label columns in „db”	Colname <—c("BIRADS", "Age", "Shape", "Margin", "Density", "outcome")
1.3) Assign factor-levels for factors (categorial variables) and set reference category	db$Margin <—as.factor (ifelse (db$Margin = = "1", "Circumscribed", ifelse (db$Margin = = "2","microbulated", ifelse (db$Margin = = "3", "Obscured", ifelse (db$Margin = = "4", "Ill-defined", "Spiculated"))))) db$Margin <—relevel (db$Margin, ref = "Circumscribed")
1.4) Assign numeric status for numerical variables	db$Age <—as.numeric (db$Age)
1.5) Replace unlogical age values as missing values	AGE_upper <—120 AGE_lower <—0 db$Age <—ifelse (db$Age > = AGE_upper, NA, db$Age) db$Age <—ifelse (db$Age < = AGE_lower, NA, db$Age)
1.6) Remove variables that have over 50% missing values	missing_col <—colMeans (is.na (db)) remove < -vector() for(i in 1:length(missing_col)){ if(missing_col[i] > = 0.5){remove < -append(remove, names(missing_col[i]))}} if(!is.logical(remove)) db < -db % > % dplyr::select(-!!remove)
1.7) Split dataset into development and validation sets	train_index <—createDataPartition(db$outcome, p = .8, list = FALSE, times = 1) db_train <—db[train_index,] db_test <—db[-train_index,]
1.8) Define recipe for data pre-processing	recipe <—recipe (outcome ~ Age + Shape + Margin + Density, data = db_train) recipe <—recipe % > % step_knnimpute(all_predictors(), neighbors = 5) % > % step_BoxCox(all_numeric(),-all_outcomes()) % > % step_other(all_nominal(), threshold = .1, other = "other") step_zv(all_predictors(),-all_outcomes()) % > % step_nzv(all_predictors(),-all_outcomes())% > % step_normalize(all_numeric(),-all_outcomes())% > % step_dummy(all_nominal(),-all_outcomes()) % > % step_corr(all_predictors(),-all_outcomes(), threshold = 0.9)
1.9) Show all steps in the recipe	prep <—prep (recipe, db_train) tidy (prep)
1.10) Examine changes of a specific pre-processing step (number 6 = normalize)	tidy (prep, number = 6)
1.11) Examine pre-processed training data	prep[["template"]]
2) Algorithm Development	
2.1) Define multiple performance metrics for model training	MySummary <—function (data, lev = NULL, model = NULL){ a1 <—defaultSummary(data, lev, model) b1 <—twoClassSummary(data, lev, model) c1 <—prSummary(data, lev, model) out <—c(a1, b1, c1) out}
2.2) Define general training parameters for cross-validation and hypergrid-search	cv <—trainControl (method = "repeatedcv", number = 10, repeats = 3, **search = "grid",** verboseIter = TRUE, classProbs = TRUE, returnResamp = "final", savePredictions = "final", summaryFunction = MySummary, selectionFunction = "tolerance", allowParallel = TRUE)
2.3) Define general training parameters for cross-validation and random grid- search	cv <—trainControl( method = "repeatedcv", number = 10, repeats = 3, **search = "random"**, verboseIter = TRUE, classProbs = TRUE, returnResamp = "final", savePredictions = "final", summaryFunction = MySummary, selectionFunction = "tolerance", allowParallel = TRUE)
2.4) Define general training parameters for adaptive resampling for hyperparameter tuing	adaptControl <—trainControl( method = "adaptive_cv", number = 10, repeats = 3, **adaptive = list(min = 5, alpha = 0.05, method = "gls", complete = TRUE),** search = "random", verboseIter = TRUE, classProbs = TRUE, returnResamp = "final", savePredictions = "final", summaryFunction = MySummary, selectionFunction = "tolerance", allowParallel = TRUE)
2.5) Hypergrid for Logistic Regression with Elastic Net Penalty	hyper_grid_glm <—expand.grid( alpha = seq(from = 0.01, to = 1, by = 0.01), lambda = seq(from = 0.01, to = 1, by = 0.01))
2.6) Hypergrid for XGBoost Tree	hyper_grid_xgboost <—expand.grid( nrounds = seq(from = 25, to = 100, by = 25), max_depth = seq(from = 5, to = 35, by = 10), eta = seq(from = 0.2, to = 1, by = 0.2), gamma = seq(from = 1, to = 10, by = 1), colsample_bytree = seq(from = 0.6, to = 1, by = 0.2), min_child_weight = seq(from = 2, to = 5, by = 1), subsample = 1)
2.7) Hypergrid for MARS algorithm	hyper_grid_mars <—expand.grid( degree = seq(from = 1, to = 3, by = 1), nprune = seq(from = 1, to = 10, by = 1))
2.8) Hypergrid for SVM with polynomial kernel	hyper_grid_svm <—expand.grid( degree = seq(from = 1, to = 11, by = 2), scale = seq(from = 0.1, to = 1, by = 0.1), C = seq(from = 0.5, to = 8, by = 0.5))
2.9) Hypergrid for multi-layer perceptron with dropout cost (deep neural network)	hyper_grid_nn <—expand.grid( size = seq(from = 1, to = 21, by = 10), dropout = seq(from = 0.1, to = 0.3, by = 0.1), batch_size = seq(from = 1, to = 11, by = 5), lr = seq(from = 0.25, to = 1, by = 0.25), rho = seq(from = 0.25, to = 1, by = 0.25), decay = seq(from = 0.1, to = 0.5, by = 0.2), cost = seq(from = 0.25, to = 1, by = 0.25), activation = 'relu')
2.10) Train Logistic Regression with Elastic Net Penalty (hypergrid search)	cv_glm <—caret::train(recipe, data = db_train, method = "glmnet", metric = "Kappa", trControl = cv, tuneGrid = hyper_grid_glm)
2.11) Train XGBoost Tree (hypergrid search)	cv_xgboost <—caret::train(recipe, data = db_train, method = "xgbTree", metric = "Kappa", trControl = cv, tuneGrid = hyper_grid_xgboost)
2.12) Train MARS algorithm (hypergrid search)	cv_mars <—caret::train(recipe, data = db_train, method = "earth", metric = "Kappa", trControl = cv, tuneGrid = hyper_grid_mars)
2.13) Train SVM with polynomial kernel (random grid search)	cv_svm <—caret::train(recipe, data = db_train, method = "svmPoly", metric = "Kappa", tuneLength = 30, trControl = cv_svm)
2.14) Train multi-layer perceptron with dropout cost (deep neural network) (random grid search)	cv_nn <—caret::train(recipe, data = db_train, method = "mlpKerasDropoutCost", metric = "Kappa", tuneLength = 30, trControl = cv_nn)
3) Internal Testing	
3.1) Show final model with optimal hyperparamters for Logistic Regression with Elastic Net Penalty	cv_glm$bestTune
3.2) Show internal testing results for final model with optimal hyperparamters (Logistic Regression with Elastic Net Penalty)	cv_glm$results[c(#bestTune),]
4) (External) Validation	
4.1) Use trained model to predict outcome probabilities in the validation set	predict (cv_glm, db_test, type = "prob")
4.2) Use trained model to predict outcome classes in the validation set	predict (cv_glm, db_test)
4.3) Calculate area under the ROC curve for outcome predictions in the validation set	roc_glm_validation = roc (as.vector (db_test$outcome), as.matrix (predict (cv_glm, db_test, type = "prob")$"Malignant")) auc_glm_validation = pROC::auc (roc_glm_validation) auc_CI_glm_validation = pROC::ci.auc (roc_glm_validation, method = "bootstrap", boot.stratified = TRUE)
4.4) Plot ROC curves	plot.roc (roc_glm_validation, legacy.axes = TRUE) lines (roc_glm_validation, col = "blue") lines (roc_xgboost_validation, col = "red") lines (roc_mars_validation, col = "orange") lines (roc_svm_validation, col = "black") lines (roc_nn_validation, col = "grey60") legend ("bottomright", legend = c("LR with Elastic Net Penalty", "XGBoost Tree", "MARS", "SVM", "neural network"), col = c("blue", "red", "orange", "black", "grey60"))
4.5) Create confusion matrix	confusionMatrix (as.factor (predict (cv_glm, db_test)), factor (db_test$outcome), positive = "Malignant")
4.6) Create calibration plot	glm_calplot_validation <—calibration(factor (db_test$outcome) ~ as.matrix (predict (cv_glm, db_test, type = "prob")$"Malignant"), data = db_test, cuts = 10) xyplot(glm_calplot_validation, auto.key = list(columns = 2))
4.7) Calculate Spiegelhalter’s Z score to assess model calibration	Spiegelhalter_z = function(y, prob){ alpha = 0.05 z_score = sum((y-prob)*(1–2*prob))/sqrt(sum(((1–2*prob)^2)*prob*(1-prob))) print(z_score) if (abs(z_score) > qnorm(1-alpha/2)){ print('reject null. NOT calibrated') } else{ print('fail to reject. calibrated') } cat('z score: ', z_score, '\n') cat('p value: ', 1-pnorm(abs(z_score)), '\n') return(z_score)} Spiegelhalter_z (unfactor(revalue(db_test$outcome, c("Malignant" = 1, "Benign" = 0))), as.matrix(predict(cv_glm, db_test, type = "prob")$"Malignant"))
4.8) Test for differences in area under the curve between two algorithms	roc.test (roc_glm_validation, roc_mars_validation, method = "bootstrap", alternative = "two.sided", boot.n = 2000, boot.stratified = TRUE)
4.9) Test for differences in diagnostic performance between two algorithms using a McNemar test	mcnemar.test (predict (cv_glm, db_test), predict(cv_mars, db_test), correct = TRUE)

Following the creation of the dataset, we convert each column to a factor (categorical) or numeric variable according to the dataset description and assign specific factor levels and reference categories to improve readability (Table 1, Task 1.3 and 1.4).

Before beginning the ML analysis, it is a good idea to go over each column to detect any variables with a high proportion of missing values or errors. Using the code in Table 1 (Task 1.6), we can detect and remove variables with a high missing rate. Although we will use imputation techniques to address missing data, we remove any variable with more than 50% of data points missing. In addition, some datasets may have data which are entered incorrectly and are either not possible or very likely to be incorrect, e.g., AGE < 0. We can use the code in Table 1 (Task 1.5) to replace these data with “NA”. It should be noted that this code is for demonstration purposes only and that there is no need to implement them in the current case.

The standard practice in ML is to use a training set for model development and a separate test set for evaluating the model's performance. Due to the high number of parameters in an ML model, overfitting the training data is a distinct possibility. Ideally, we have a completely independent dataset (e.g., which contains data collected from a different practice or hospital) to validate our model. Alternatively, if we only have one dataset, we can randomly split our dataset into a development set, which contains 80% of the data, and a validation set, which contains the remaining 20% (Table 1, Task 1.7).

#### Data pre-processing

**Fig. 1:**
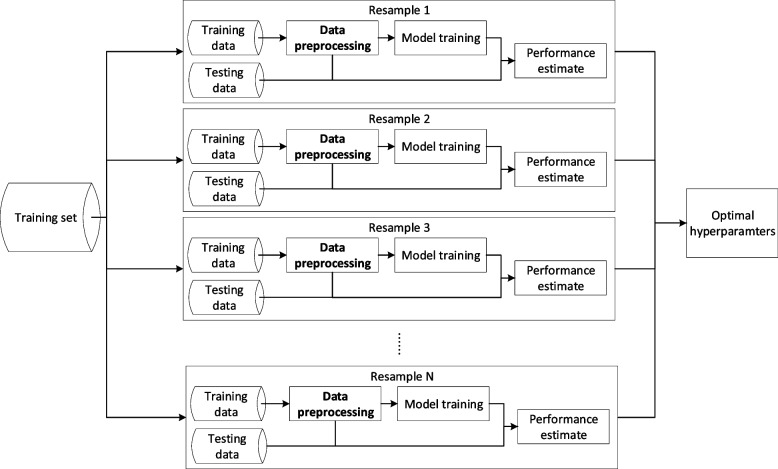
Data pre-processing within the resampling process

Now, we begin to pre-process the data. Data pre-processing should be performed within each isolated resampling iteration (Fig. 1) to avoid information leakage which happens when models are trained using information from outside the training dataset and which reduces model generalizability to future data [14, 18]. The sequence of the data pre-processing steps has implications on the model output, too. For instance, conducting one-hot encoding before normalization may change the information of the data and generate misleading models. A thorough discussion regarding information leakage during data pre-processing and the order of data pre-processing techniques is referenced for interested readers [14].

The `recipes` package available in R offers an elegant way to create a blueprint (recipe) containing various steps of data pre-processing. We will create a blueprint that will be applied separately on each fold during the cross-validation (CV) process to minimize information leakage. We first define a recipe object with our training dataset and variable information in a formula format (Table 1, Task 1.8). Then, we go through a series of steps to sequentially add data pre-processing techniques needed to prepare the training data using the recipe. Table 2 provides a description of all pre-processing steps used in this case study. The "all_predictors()" function is used to denote that we apply this step to all predictors rather than a subset of predictors. Other alternatives to all_predictors() include "all_numerics()", used for numeric variables, and "all_nominals()" for categorical variables (Table 1, task 1.8). We can also use predictor names to specify where the steps should be applied. For example, “step_log(AGE)” would return log-transformed versions of the AGE variable. By contrast, the "-all_outcomes()" is used to exclude the outcome variable from the step. Based on our experience, these steps are sufficient for many medical datasets. We suggest that careful consideration is given about which steps to use and their sequence based on the dataset at hand. A more in-depth discussion on data pre-processing and feature engineering techniques is published elsewhere [14]. Readers requiring additional data pre-processing steps are directed to the `recipes` package's website for a full list of functions [19].

**Table 2:** Description of data pre-processing steps

Step	Description
step_knnimputation	Impute missing values using the *k*-nearest neighbor algorithm
step_BoxCox	Transform numeric data using simple Box-Cox transformation
step_other	Pool less frequent categories into an "other" category for categorical variables
step_zv	Remove variables that have a single value
step_nzv	Remove variables having the frequency ratio of their first and second frequent values above 95/5 and the number of unique values over the total number of samples below 10%
step_normalize	Normalize numeric variables to have zero mean and one unit of variance (standard deviation = 1)
step_dummy	Covert each level of categorical variables into a numeric binary term
step_corr	Remove variables that are highly correlated with other variables (absolute correlation values > = 0.9)

*Note*. A thorough introduction to each step can be found in the package document “recipes” [20]

**Fig. 2:**
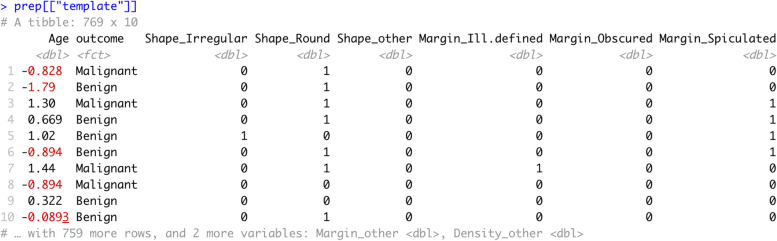
Excerpt training dataset after pre-processing steps

We recommend looking at the prepared data before fitting ML algorithms to the data: First, we have a look at all steps included in the recipe (Table 1, Task 1.9). Second, we can examine the changes of a specific pre-processing step, e.g., the mean and standard deviation for step_normalize (Table 1, Task 1.10; interested readers can modify the value of the "number" to examine other steps in the blueprint). Third, we review the prepared training data altogether (Table 1, Task 1.11) – an excerpt of the pre-processed training data is shown in Fig. 2.

#### Algorithm development and hyperparameter tuning

After having prepared the dataset, we can start developing our models. Before getting to the different model algorithms, we first define some general settings that can be used for all algorithms. First, we define the performance metrics that will be used to select the final model during the training process. For this purpose, we write a summary function (Table 1, Task 2.1) that contains multiple performance metrics like AUROC, accuracy, sensitivity, or Kappa that will all be calculated during the training process and of which we can later choose one metric to select the final model.

Next, we define some general training parameters that determine how each model will be trained (Table 1, 2.2). To train our models, we use tenfold cross-validation with three repetitions and a grid search to determine the optimal combination of model hyperparameters. This means that our development set will be split into ten equal subsamples (“folds”), of which nine are used to train a model and 1 to test the models’ performance. This method is adopted to avoid performance fluctuation due to the random subsampling: the process is repeated ten times so that each of the ten subsamples is used exactly one time as a test set. The whole tenfold cross-validation procedure will then be repeated three times to further improve the generalizability of our models (the ten random subsamples will be different every time). This results in a total of 30 models being developed and tested for each specific combination of model hyperparameters. In order to determine the optimal hyperparameters, the whole cross-validation process will be performed for every possible combination of hyperparameters as specified in a hyperparameter grid (see model-specific hyperparameter grids in Table 1, Task 2.4 to 2.8). Depending on how large and granular our hyperparameter grid is, we can easily end up developing over 1,000 models which explains the high computational requirements for this element of ML. The performance of these models will be evaluated using the performance metrics we defined above (“MySummary”).

Finally, we have to define which combination of hyperparameters will be chosen as the final model (as determined by the average performance on the resampled test sets). Choosing the model that achieved the highest performance is common but can result in bad generalizability when we (externally) validate the model. Thus, we use a tolerance threshold which means that the simplest model that is within a given percentage (default: 3%) tolerance of the empirically optimal model will be chosen as the final model [21].

After defining the general performance measures and training settings, we come to the ML algorithms. We will develop five different algorithms: Logistic Regression (LR) with Elastic Net penalty, Extreme Gradient Boosting (XGBoost) tree, Multivariate Adaptive Regression Spline (MARS), a Support Vector Machine (SVM) with polynomial kernel, and a deep neural network (multi-layer perceptrons). Each algorithm has unique hyperparameters that we have to tune during the training process. For illustration purposes, we will discuss the necessary steps for the LR with Elastic Net Penalty – the “R” code for all algorithms is given in Table 1 (Tasks 2.10 to 2.14). For the LR with Elastic Net Penalty, there are two hyperparameters, alpha and lambda. We create a hyperparameter grid that contains possible combinations of these two parameters (10,000 combinations; Table 1, Task 2.4).

Next, we can specify the exact algorithm and data on which we would like to train our algorithm (Table 1, Task 2.10): We apply our previously prepared “recipe” to our development dataset as well as the previously defined parameters for the cross-validation process (“cv”) and hyperparameter grid (“hyper_grid_glm”). Moreover, we select the specific algorithm (“glmnet”), and we select one performance metric from the performance metrics we defined to select our final model (the “Kappa” metric is helpful for imbalanced datasets as it takes the observed as well as the expected agreement between predictions and ground truth outcomes into account, another common choice is “ROC” for the area under the ROC curve).

For more complex algorithms like the SVM or the neural network, determining the optimal hyperparameter values by evaluating the model performance for every possible combination in a hyperparameter grid can be very time-consuming. An alternative approach is to perform a random search of optimal hyperparameters, which has been shown to only minimally affect the algorithm performance [22]. To perform a random hyperparameter search, we have to adapt our general training parameters and replace the “grid” with “random” (Table 1, Task 2.3). Current research focuses on developing new approaches for hyperparameter optimization, like Bayesian hyperparameter optimization, evolutionary algorithms, or adaptive resampling [23–25]. The common goal of these approaches is to reduce computational burden compared to a full hyper-grid search but maintain predictive performance. As an example, the code to conduct hyperparameter optimization via adaptive resampling is shown in Table 1, Task 2.4.

#### Internal testing using resampling methods

**Fig. 3:**
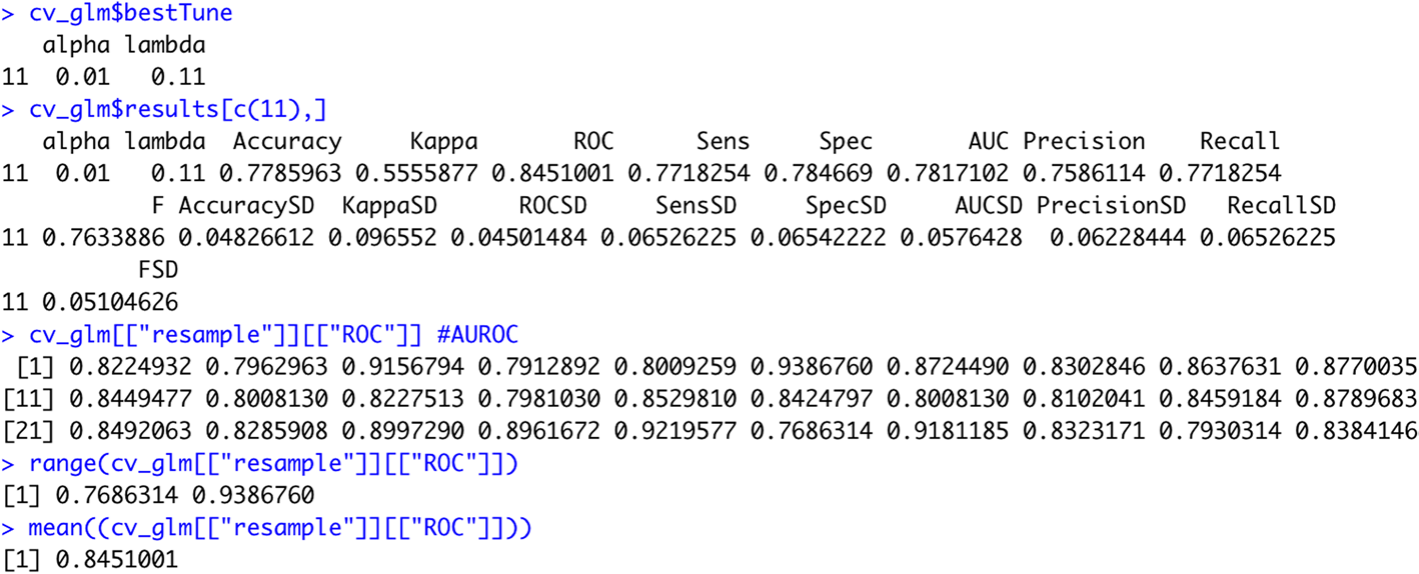
Final model and internal testing results for the Logistic Regression with Elastic Net Penalty

After having our models trained, we can have a look at the final model that was chosen during the resampling process and its hyperparameters and performance during the resampling process (Table 1, Tasks 3.1 and 3.2). Figure 3 shows the exemplary output we receive for the LR with Elastic Net Penalty: we can see that the final hyperparameter values were determined as alpha = 0.01 and lambda = 0.11. This hyperparameter combination had an AUROC of 0.85. If we take a closer look into the model specifications, we can see that this value of 0.85 is the mean value of the 30 models that were developed for this combination of hyperparameters (tenfold cross-validation with three repetitions) – the AUROC values of those 30 models ranged from 0.77 to 0.94 which illustrates the importance of the repeated resampling process to avoid coincidentally choosing the hyperparameter combinations that perform well only on a specific subsample.

#### External validation

In this step, we will evaluate the performance of our final, tuned model on a validation dataset. Some of the techniques we show here, like confusion matrices and model calibration, can be easily applied to the internal testing results as well. In general, to evaluate the model performance, we use the trained model to predict outcome probabilities or outcome classes in the validation set (Table 1, Tasks 4.1 and 4.2) and then compare those predictions with the actually observed outcomes.

**Fig. 4:**
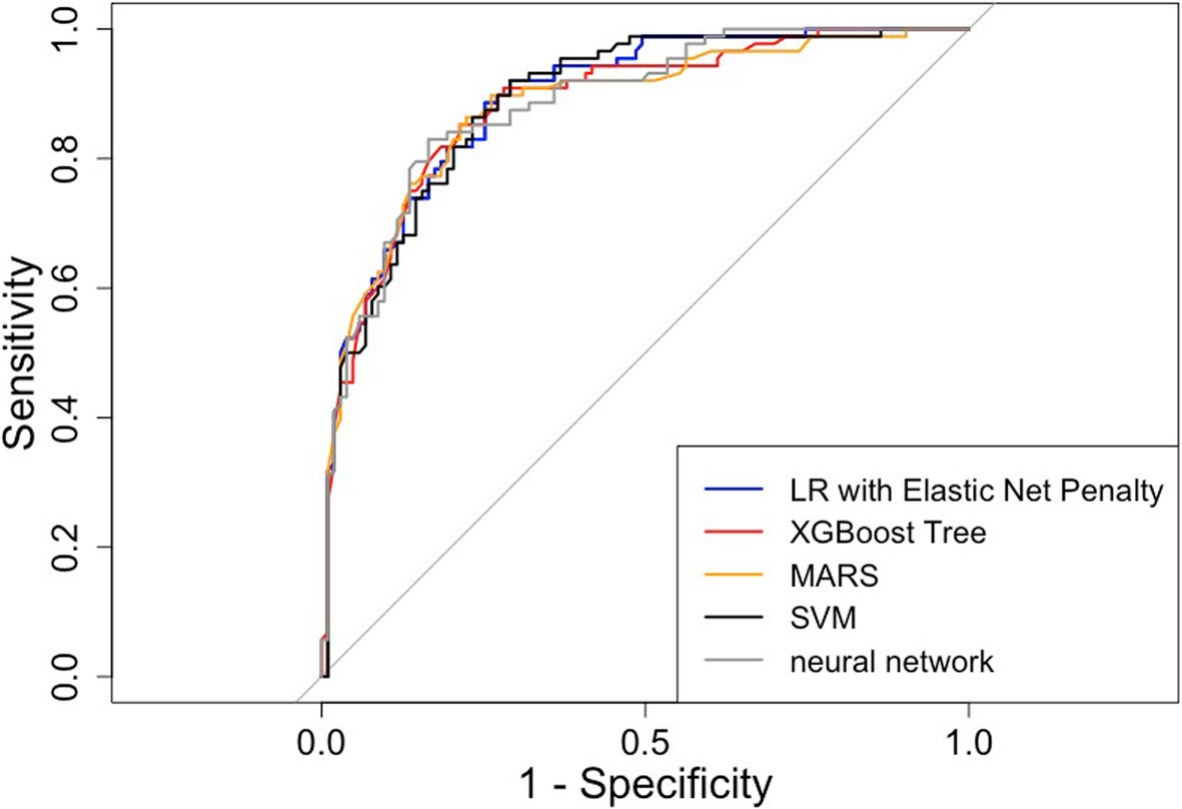
Receiver Operating Characteristic curves in the Validation Set

At first, we perform a ROC analysis by comparing the outcome predictions of our previously-developed model in the validation set with the actual outcomes in the validation set; AUROC and 95% confidence intervals are computed using 2000 stratified bootstrap replicates (Table 1, Task 4.3). AUROC for the LR with elastic net penalty was 0.89 (95% CI 0.85 – 0.94), for the XGBoost Tree 0.88 (95% CI 0.83 – 0.93), for the MARS algorithm 0.88 (95% CI 0.83 – 0.93), for the SVM 0.89 (95% CI 0.84 – 0.93), and for the neural network 0.89 (95% CI 0.84 – 0.93). Accompanying ROC curves are shown in Fig. 4, and the accompanying R code is listed in Table 1 (“Plot ROC curves”).

For a more detailed performance evaluation, we compute confusion matrices and accompanying diagnostic metrics like sensitivity, specificity, and negative- and positive-predictive values (Table 1, Task 4.5). While the AUROC provides an important overall metric of model performance, only confusion matrices and derived diagnostic performance metrics can provide an actual, clinically meaningful interpretation of a diagnostic test. As an example, Table 3 shows the confusion matrix of the MARS algorithm in the validation set: of the 191 patients in the validation set, the MARS algorithm classified 79 breast lesions correctly as malignant (true positives), 75 correctly as benign (true negative), 28 falsely as malignant (false-positive), and 9 falsely as benign (false-negative), resulting in a sensitivity of 89.8% and a specificity of 72.8%. Moreover, the confusion matrix provides information on whether significant classification improvement has been achieved: The so-called “no information rate” refers to the proportion that the most frequent class has. In our case, benign breast lesions (103 of 191, 53.9%) are more frequent than malignant lesions (88 of 191, 46.1%). Thus, the accuracy of a classification algorithm should be significantly higher than 53.9%, i.e., the classification algorithm should result in a significant improvement over the no information rate. In our case, the accuracy of the MARS algorithms is significantly higher than the non information rate (81.2 vs. 53.9%, *p* < 0.001).

**Table 3:** Confusion Matrix and Diagnostic Performance Metrics for Multivariate Adaptive Regression Spline Algorithm

	Reference	
Prediction	Malignant	Benign
Malignant	79	28
Benign	9	75
Sensitivity	89.8% (79 of 88)	
Specificity	72.8% (75 of 103)	
Negative predicitive value	89.3% (75 of 84)	
Positive predictive value	73.8% (79 of 107)	
Accuracy	81.2% (155 of 191)	
No information rate	0.54	
*p*-Value [Acc > NIR]*	< 0.001	

^*^Refers to a one-sided binomial test determining whether the accuracy proportion is higher than the no-information rate

**Fig. 5:**
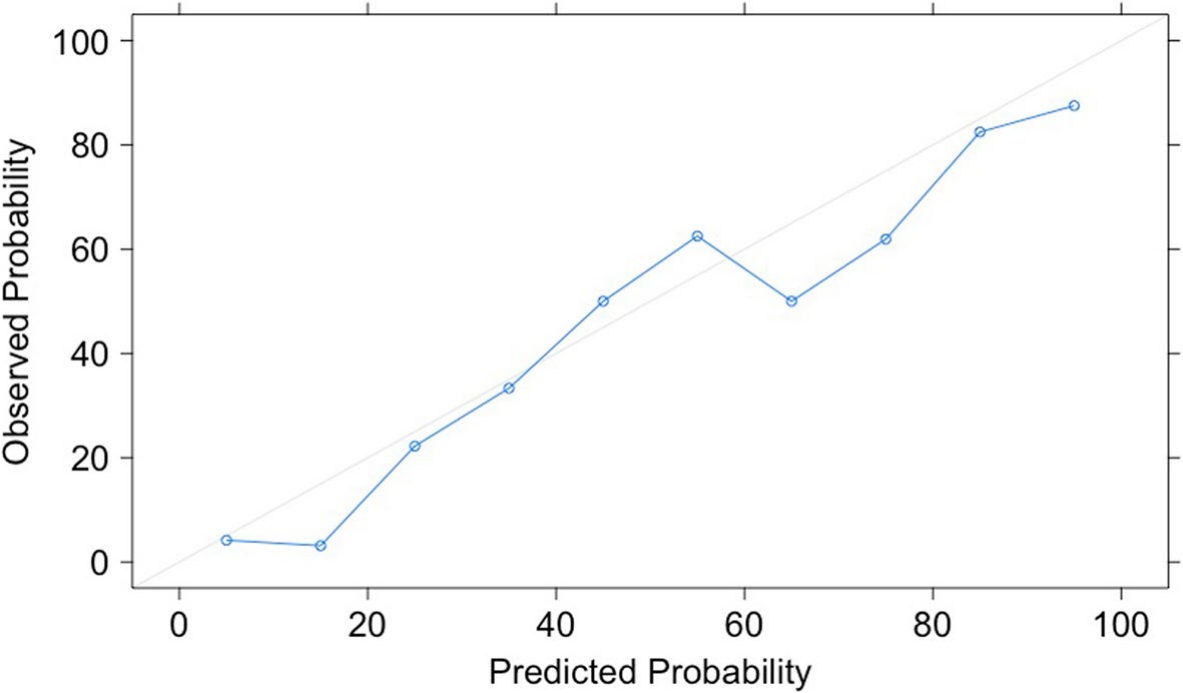
Calibration Plot of the Support Vector Machine

To evaluate model calibration, we compute calibration plots (observed vs. predicted probabilities) and Spiegelhalter’s Z statistic [26, 27]. Taking the SVM algorithm as an example and using the code shown in Table 1 (Tasks 4.6 and 4.7), the calibration plot yields a well-calibrated model (Fig. 5) which is confirmed by Spiegelhalter’s Z (z score: -0.337, *P* value: 0.368).

### Fit analysis

The goodness of fit analyses are used to evaluate how well a model fits actual data. Conventional goodness of fit tests for binary outcome models, like the Chi-Square test or Hosmer–Lemeshow test, are not always recommended for ML models: They tolerate little disagreement between model predictions and observed outcomes with large sample size, and the arbitrary choice of group numbers is also problematic as ML algorithms are often applied to high-dimensional datasets with large numbers of covariates [28, 29]. Testing for differences between the accuracy and the no-information rate has been suggested previously as well. Thus, fit analyses for ML models are usually conducted by comparing model performance between training and testing sets with three potential outcomes.

1. Model performance in the training set is relevantly higher compared to the testing set. This indicates that the model is overfitted to the training data, and model complexity needs to be reduced.
2. Model performance in the training and testing set is bad. This indicates that the model is under-fitted, and model complexity can be increased by, for example, adding additional variables.
3. Model performance in the training and testing set is good. This indicates a good model fit.

Table 4 illustrates the comparison of model performance across training and testing sets for the MARS algorithm. Discrimination performance is evaluated by AUROC and accuracy for this binary outcome model (residual-mean-square errors would be appropriate for linear outcome models) and calibration performance by Spiegelhalter’s Z score. While Spiegelhalter’s Z-score provides a way to test ML model calibration, the role of Chi-Square tests for goodness of fit assessment for high-dimensional ML datasets is yet unclear. However, both tests indicate a good model fit (keep the null hypothesis specifying no significant difference between the observed and the expected values) as does the significantly higher accuracy compared to the no-information rate.

**Table 4:** Discrimination and Calibration Performance in the Training and Testing Sets for Multivariate Adaptive Regression Spline Algorithm

	Training set (cross-validation)	Testing set
Discrimination performance		
AUROC	0.84 (95% CI 0.82–0.86)	0.88 (95% CI 0.83–0.93)
Accuracy	77.6% (95% CI 75.6%—79.5%)	81.2% (95% 74.9%—86.4%)
No-information rate	53.7%	53.9%
* p*-value [accuracy > no-information rate]*	< 0.001	< 0.001
* p*-Value [Chi-Square goodness of fit]	0.10	0.08
Calibration performance		
Spiegelhatler’s Z-score	0.83	-1.31
* p*-value	0.20	0.09

^*^Refers to a one-sided binomial test determining whether the accuracy proportion is higher than the no-information rate

### Model comparison

Finally, we compare the performance among algorithms and test for statistically significant differences. We use two standard approaches: the McNemar test to test for differences in the distribution of disagreement between two algorithms and bootstrap replicates to compare the AUROC values. Table 1 (Task 4.8) shows the code to compare the AUROC of the LR with elastic net penalty and the MARS algorithm using 2000 bootstrap replicates drawn from the validation set and stratified for the outcome variable. The *P* value of 0.240 indicates that the AUROC of the two algorithms does not differ significantly (AUROC 0.89 vs. 0.88) in the validation set. Additionally, the results of the McNemar test (*P* = 0.773) indicate that there are no significant differences in algorithm performance (Table 1, Task 4.9).

### Ethical considerations

The research reported in this article complies with the Declaration of Helsinki. For this analysis, we used de-identified data from a public repository [16]. As such, ethical approval was not required.

## Results

**Fig. 6:**
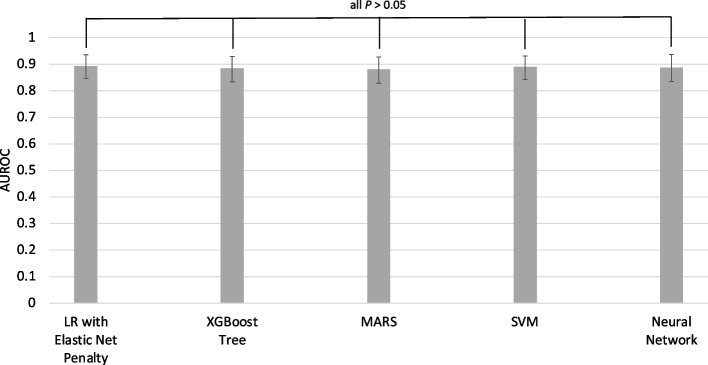
Performance Comparison – Differences in Area under the Curve

The five algorithms showed equally high performance in the validation set (*n* = 191) to classify breast masses as benign or malignant based on mammography image features and patient age. AUROC for the LR with elastic net penalty was 0.89 (95% CI 0.85 – 0.94), for the XGBoost Tree 0.88 (95% CI 0.83 – 0.93), for the MARS algorithm 0.88 (95% CI 0.83 – 0.93), for the SVM 0.89 (95% CI 0.84 – 0.93), and for the neural network 0.89 (95% CI 0.84 – 0.93). When comparing the five algorithms against each other, AUROC did not differ significantly (Fig. 6).

## Discussion

In this work, we expanded our previous introductory guide to ML in medicine by providing a best-practice example of some ML techniques like data pre-processing, hyperparameter tuning, and model comparison. We used open-source software and a publicly available dataset to train and validate multiple ML models to classify breast masses using mammography image features with exceptional performance. The full analysis code is shown in the Supplementary Appendix. Our paper allows (medical) researchers who are interested in using ML algorithms for their analyses to perform a comprehensive ML analysis on their own.

After stoking great enthusiasm from the community, medical ML applications are currently undergoing a critical reality check [11]. Incidents like Google’s AI software to automatically diagnose diabetic retinopathy from fundus images, which showed great performance on retrospectively retrieved clinical data but proved impractical upon real-world application in Thailand, contribute to deteriorating trust in medial ML applications [30, 31]. This reproducibility crisis of medical ML applications has also been observed for models used to diagnose or predict outcomes related to the Covid-19 pandemic and short-term oncologic outcomes [10, 32]. A high risk of bias for these models due to inappropriate validation techniques and unstandardized or unclear approaches regarding hyperparameter tuning was identified in these studies. Following our instructions for data pre-processing, hyperparameter tuning, and model comparison may help to improve model generalizability and reproducibility and thus to build trust in medical ML applications.

Data pre-processing, hyperparameter tuning, and proper model comparison are essential steps to improve model performance, generalizability, and evaluation. Data pre-processing accounts for about 80% of data analysis efforts and has significant impacts on model outputs [33]. For ML in medicine, data preparation is especially critical due to the use of electronic health record (EHR) data. Although EHR data is the most suitable and valuable source for model development as it reflects real-world practices, the data quality is low compared to data from trials and registration repositories. Models trained with low-quality data may have a suboptimal performance and provide clinical decision-makers with misleading information causing unintentional harm [34]. In the current paper, we demonstrated a proper implementation of data pre-processing in the ML pipeline to maximize its benefits and prevent unintended information leakage during the processes.

When interpreting the overall model performance, statistical tests are important to evaluate whether differences in model performance are actually statistically significant. Many articles on ML in medicine simply compare the values of performance metrics without actually testing for differences [35]. For example, in our analysis, we cannot conclude that the XGBoost Tree performs worse compared to the neural network (AUROC 0.88 vs. 0.89) because this difference is not statistically significant (*P*> 0.05). We highly encourage medical researchers who use ML to statistically test for actual differences to ensure a fair and comprehensive evaluation of the models they train. Moreover, the choice of evaluation metric may depend on the clinical scenario. For example, current research in the area of breast cancer treatment focuses around the reliable exclusion of residual cancer after neoadjuvant chemotherapy for the possible omission of breast surgery [36]. In this scenario, sensitivity is prioritized over specificity, and recent advancements in this field suggest that ML algorithms with high sensitivity may be able to reliably exclude residual cancer, pending further validation [37].

Our present manuscript expands on our previous introductory paper on ML in medicine to explain techniques like data pre-processing, hyperparameter tuning, and model comparison. For the purpose of this educational paper, we chose a publicly available dataset to ensure easy access and reproducibility. This choice of dataset comes, however, with some limitations. First, the sample size (961 records) is quite small and likely results in biased models. As our manuscript intends to provide a standardized methodological approach developing ML models for interested medical researchers, we do not perceive this to be a limitation for this specific aim. However, we would like to clearly note that any developed ML algorithm should be prospectively validated before clinical implementation. Second, the number of predictive features is limited to patient age and some mammography image features. This does not reflect the current clinical routine in breast cancer diagnostics which includes many more factors like ultrasound, MRI, family medical history, genomic information, and many more. Current evidence suggests that multi-modal imaging and clinical information are highly relevant for AI-based algorithms when classifying breast masses [38]. Although limited types of variable is not perceived as a limitation for this study given its purpose, we argue that transdisciplinary work between clinical and methodology partners to enable additional data types is of utmost importance.

Moreover, there are many other cutting-edge issues in the field of ML that are not covered yet in the present analysis: Frequent racial bias by self-learning algorithms has been identified as a major concern in the field of ML [39], imbalanced datasets because of rare outcome events (e.g., early-stage breast cancer mortality) are a major challenge for self-learning algorithms in many medical disciplines; Providing insights into black-box model predictions by using model-specific or model-agnostic interpretations is an emerging field of research [40, 41]; and Unsuccessful implementation of digital health tools due to various factors, e.g., lack of transdisciplinary knowledge, is a major issue [42, 43]. We hope to expand our series on ML in medicine to cover some of these aspects in the near future.

## Conclusions

Our paper allows medical science researchers interested in using ML algorithms for their analyses to perform a comprehensive ML analysis on their own, including techniques like data pre-processing, hyperparameter tuning, and model comparison. Following our instructions may help to improve model generalizability and reproducibility and thus to build trust in medical ML applications.

## Supplementary Information

**Additional file 1.**

## Data Availability

The datasets generated and/or analyzed during the current study are available in the University of California, Irvine Machine Learning Repository (“Mammographic Mass” dataset), https://archive.ics.uci.edu/ml/datasets/Mammographic+Mass. The source code used for data analysis is provided in the supplement of this article.

## Abbreviations

AUC: Area Under the Receiver-Operating Characteristic Curve
CI: Confidence Interval
ML: Machine learning
ROC: Receiver-Operating Characteristic
SVM: Support Vector Machine
XGBoost Tree: Exterme Gradient Boosting Tree

## References

[1] Yu KH, Beam AL, Kohane IS (2018). Artificial intelligence in healthcare. Nat Biomed Eng.

[2] Scott IA (2018). Machine learning and evidence-based medicine. Ann Intern Med.

[3] Rajkomar A, Dean J, Kohane I (2019). Machine learning in medicine. N Engl J Med.

[4] Hosny A, Parmar C, Quackenbush J, Schwartz LH, Aerts HJWL (2018). Artificial intelligence in radiology. Nat Rev Cancer.

[5] Pfob A, Mehrara BJ, Nelson JA, Wilkins EG, Pusic AL, Sidey-Gibbons C. Towards Patient-Centered Decision-Making in Breast Cancer Surgery. Ann Surg 2021; published online March 18. 10.1097/SLA.0000000000004862. 10.1097/SLA.0000000000004862 PMC9762704 33914464

[6] Pfob A, Sidey-Gibbons C, Lee HB (2021). Identification of breast cancer patients with pathologic complete response in the breast after neoadjuvant systemic treatment by an intelligent vacuum-assisted biopsy. Eur J Cancer.

[7] Sidey-Gibbons C, Pfob A, Asaad M (2021). Development of machine learning algorithms for the prediction of financial toxicity in localized breast cancer following surgical treatment. JCO Clin Cancer Inform.

[8] Liu X, Cruz Rivera S, Moher D, et al. Reporting guidelines for clinical trial reports for interventions involving artificial intelligence: the CONSORT-AI extension. Lancet Digit Heal 2020;0. 10.1016/S2589-7500(20)30218-1. 10.1016/S2589-7500(20)30218-1 PMC8183333 33328048

[9] Cruz Rivera S, Liu X, Chan A-W, et al. Guidelines for clinical trial protocols for interventions involving artificial intelligence: the SPIRIT-AI extension. Lancet Digit Heal 2020;0. 10.1016/S2589-7500(20)30219-3. 10.1016/S2589-7500(20)30219-3 PMC8212701 33328049

[10] Roberts M, Driggs D, Thorpe M (2021). Common pitfalls and recommendations for using machine learning to detect and prognosticate for COVID-19 using chest radiographs and CT scans. Nat Mach Intell.

[11] Wilkinson J, Arnold KF, Murray EJ, et al. Time to reality check the promises of machine learning-powered precision medicine. Lancet Digit Heal 2020;0. 10.1016/S2589-7500(20)30200-4. 10.1016/S2589-7500(20)30200-4 PMC9060421 33328030

[12] Sidey-Gibbons JAM, Sidey-Gibbons CJ (2019). Machine learning in medicine: a practical introduction. BMC Med Res Methodol.

[13] Harrison CJ, Sidey-Gibbons CJ (2021). Machine learning in medicine: a practical introduction to natural language processing. BMC Med Res Methodol.

[14] Boehmke B, Greenwell B. Feature & Target Engineering. In: Hands-On Machine Learning. New York: R. Packt Publishing; 2020.

[15] Alpaydin E (2020). Introduction to Machine Learning.

[16] UCI Machine Learning Repository: Mammographic Mass Data Set. available from: http://archive.ics.uci.edu/ml/datasets/mammographic+mass.

[17] Elter M, Schulz-Wendtland R, Wittenberg T (2007). The prediction of breast cancer biopsy outcomes using two CAD approaches that both emphasize an intelligible decision process. Med Phys.

[18] Samala RK, Chan H, Hadjiiski L, Helvie MA (2021). Risks of feature leakage and sample size dependencies in deep feature extraction for breast mass classification. Med Phys.

[19] Kuhn M, Wickham H. recipes. 2020. https://recipes.tidymodels.org/index.html.

[20] Kuhn M, Wickham H (2021). Package ‘recipes’.

[21] Kuhn M (2020). Classification and Regression Training - The ‘Caret’ Package.

[22] Bergstra J, Bengio Y (2012). Random Search for Hyper-Parameter Optimization Yoshua Bengio. J Mach Learn Res.

[23] Klein A, Falkner S, Bartels S, Hennig P, Hutter F (2017). Fast bayesian hyperparameter optimization on large datasets. Electron J Stat.

[24] Zitzler E, Deb K, Thiele L (1991). Comparison of Multiobjective Evolutionary Algorithms: Empirical Results. Massachusetts Inst Technol Evol Comput.

[25] Kuhn M. Futility Analysis in the Cross-Validation of Machine Learning Models. 2014; published online May. 10.48550/arxiv.1405.6974.

[26] Spiegelhalter DJ (1986). Probabilistic prediction in patient management and clinical trials. Stat Med.

[27] Harrell FE, Lee KL, Mark DB (1996). Multivariable prognostic models: Issues in developing models, evaluating assumptions and adequacy, and measuring and reducing errors. Stat Med.

[28] Nattino G, Pennell ML, Lemeshow S (2020). Assessing the goodness of fit of logistic regression models in large samples: a modification of the Hosmer-Lemeshow test. Biometrics.

[29] Huang Y, Li W, Macheret F, Gabriel RA, Ohno-Machado L (2020). A tutorial on calibration measurements and calibration models for clinical prediction models. J Am Med Informatics Assoc.

[30] Gulshan V, Peng L, Coram M (2016). Development and validation of a deep learning algorithm for detection of diabetic retinopathy in retinal fundus photographs. JAMA - J Am Med Assoc.

[31] Beede E, Baylor E, Hersch F (2020). A Human-Centered Evaluation of a Deep Learning System Deployed in Clinics for the Detection of Diabetic Retinopathy. Conference on Human Factors in Computing Systems - Proceedings.

[32] Lu SC, Xu C, Nguyen CH, Geng Y, Pfob A, Sidey-Gibbons C (2022). Machine Learning–Based Short-Term Mortality Prediction Models for Patients With Cancer Using Electronic Health Record Data: Systematic Review and Critical Appraisal. JMIR Med Inf.

[33] Zhang S, Zhang C, Yang Q (2003). Data preparation for data mining. Appl Artif Intell.

[34] Obermeyer Z, Emanuel EJ (2016). Predicting the Future — Big Data, Machine Learning, and Clinical Medicine. N Engl J Med.

[35] Pfob A, Sidey-Gibbons C, Heil J. Response Prediction to Neoadjuvant Systemic Treatment in Breast Cancer—Yet Another Algorithm? JCO Clin Cancer Informatics 2021;654–5. 10.1200/CCI.21.00033 34110930

[36] Heil J, Kuerer HM, Pfob A (2020). Eliminating the breast cancer surgery paradigm after neoadjuvant systemic therapy: current evidence and future challenges. Ann Oncol.

[37] Pfob A, Sidey-Gibbons C, Rauch G (2022). Intelligent Vacuum-Assisted Biopsy to Identify Breast Cancer Patients with Pathologic Complete Response (ypT0 and ypN0) after Neoadjuvant Systemic Treatment for Omission of Breast and Axillary Surgery. J Clin Oncol.

[38] Pfob A, Sidey-Gibbons C, Barr RG (2022). The importance of multi-modal imaging and clinical information for humans and AI-based algorithms to classify breast masses (INSPiRED 003): an international, multicenter analysis. Eur Radiol.

[39] Obermeyer Z, Powers B, Vogeli C, Mullainathan S (2019). Dissecting racial bias in an algorithm used to manage the health of populations. Science (80-).

[40] Ribeiro MT, Singh S, Guestrin C (2016). Model-Agnostic Interpretability of Machine Learning.

[41] Ribeiro MT, Singh S, Guestrin C (2016). ‘Why Should I Trust You?’: Explaining the Predictions of Any Classifier.

[42] Greenhalgh T, Wherton J, Papoutsi C, et al. Beyond adoption: A new framework for theorizing and evaluating nonadoption, abandonment, and challenges to the scale-up, spread, and sustainability of health and care technologies. J Med Internet Res 2017;19. 10.2196/jmir.8775. 10.2196/jmir.8775 PMC5688245 29092808

[43] Pfob A, Sidey-Gibbons C, Schuessler M, et al. Contrast of Digital and Health Literacy Between IT and Health Care Specialists Highlights the Importance of Multidisciplinary Teams for Digital Health—A Pilot Study. JCO Clin Cancer Informatics 2021;734–45. 10.1200/CCI.21.00032 34236897

