# Supplementary material for: Machine learning in medicine: a practical introduction to techniques for data pre-processing, hyperparameter tuning, and model comparison
Source: BMC Med Res Methodol. 2022 Nov 1;22:282. doi: 10.1186/s12874-022-01758-8 (PMC9624048; doi:10.1186/s12874-022-01758-8)
Supplement: Supplementary file 1 — Additional file 1. [file 12874_2022_1758_MOESM1_ESM.docx]

Online Supplementary Appendix

Machine learning in medicine: a practical introduction to techniques for data pre-processing, hyperparameter tuning, and model comparison

Box 1. Complete R code for data preparation and model development, performance evaluation, and comparison.

| #### (1) Install and load relevant packages ####  require(RTextTools)  require(tm)  require(SnowballC)  require(glmnet)  require(NLP)  require(SparseM)  require(rpart)  require(randomForest)  require(nnet)  require(e1071)  require(ROCR)  require(gmodels)  require(wordcloud)  require(dplyr)  require(methods)  require(pROC)  require(e1071)  require(rpart)  require(readxl)  require(foreach)  require(caret)  require(tensorflow)  require(Rfast) ###issue with ROC?  require(keras)  require(reticulate)  require(shapper)  require(DALEX)  require(xgboost)  require(tidyverse)  require(SHAPforxgboost)  require(lime)  require(rsample)  require(kernlab)  require(gmodels)  require(Amelia)  require(mlbench)  require(corrplot)  require(earth)  require(rpact)  require(gmodels)  require(varhandle)  require(plyr)  require(recipes)  require(doParallel)  require(MLmetrics )  require(Rmisc)  require(doParallel)  require(DALEX)  require(pdp)  require(vctrs)  library(mlbench)  library(tidymodels) # packages for modeling and statistical analysis  library(tune) # For hyperparemeter tuning  library(workflows) # streamline process  #### (2) Load data from UCI website ####  db<- read.csv(url("https://archive.ics.uci.edu/ml/machine-learning-databases/mammographic-masses/mammographic_masses.data"))  colname<- c("BIRADS", "Age", "Shape", "Margin", "Density", "outcome")  colnames(db)<-colname  summary(db)  #### (3) define variable types ####  sapply(db, class)  #relevel the categorical variables  db$Shape <- as.factor(ifelse(db$Shape=="1", "Round",  ifelse(db$Shape=="2", "Oval",  ifelse(db$Shape == "3", "Lobular","Irregular"))))  levels(db$Shape)  db$Shape <- relevel(db$Shape, ref = "Oval")  levels(db$Shape)  db$Margin <- as.factor(ifelse(db$Margin=="1", "Circumscribed",  ifelse(db$Margin=="2","microbulated",  ifelse(db$Margin=="3", "Obscured",  ifelse(db$Margin=="4", "Ill-defined", "Spiculated")))))  levels(db$Margin)  db$Margin <- relevel(db$Margin, ref = "Circumscribed")  levels(db$Margin)  db$Density <-as.factor(ifelse(db$Density=="1","high",  ifelse(db$Density=="2", "iso",  ifelse(db$Density=="3", "low", "fat-containing"))))  levels(db$Density)  db$Density <- relevel(db$Density, ref = "fat-containing")  levels(db$Density)  db$outcome<-as.factor(ifelse(db$outcome==1, "Malignant", "Benign"))  levels(db$outcome) #caret uses the first level of the outcome variable as reference during the training process - so first level should be the positive outcome  db$outcome <- factor(db$outcome, levels=rev(levels(db$outcome)))  levels(db$outcome)  db$Age<-as.numeric(db$Age)  db$BIRADS<-as.numeric(db$BIRADS)  # Replace AGE >120, or <0 with NA  AGE_upper <- 120  AGE_lower <- 0  db$Age <- ifelse(db$Age >= AGE_upper, NA, db$Age)  db$Age <- ifelse(db$Age <= AGE_lower, NA, db$Age)  #removing variables that have over 50% NULL rate  missing_col<-colMeans(is.na(db))  remove<-vector()  for(i in 1:length(missing_col)){  if(missing_col[i]>=0.5){  remove<-append(remove, names(missing_col[i]))  }  }  if(!is.logical(remove)) db<-db %>% dplyr::select(-!!remove)  #split dataset  my_seed = 2021  set.seed(my_seed)  train_index <- createDataPartition(db$outcome, p = .8,  list = FALSE,  times = 1)  db_train <- db[train_index,]  db_test <- db[-train_index,]  CrossTable(db$outcome)  CrossTable(db_train$outcome)  CrossTable(db_test$outcome)  #### (4) define blueprint for data pre-processing ####  library(recipes)  predictors<- c("Age", "Shape", "Margin", "Density")  outcome<-"outcome"  recipe<-recipe(outcome ~ Age + Shape + Margin + Density, data = db_train)  recipe <- recipe %>%  step_knnimpute(all_predictors(), neighbors = 5) %>% # impute with KNN  step_BoxCox(all_numeric(),-all_outcomes()) %>% # boxcox transformation for all numeric features  step_other(all_nominal(), threshold = .1, other = "other") %>% # (all nominal selects all factors): lumping: If it's less than one then factor levels whose rate of occurrence in the training set are below threshold will be "othered". If it's greater or equal to one then it's treated as a frequency and factor levels that occur less then threshold times will be "othered".  step_zv(all_predictors(),-all_outcomes()) %>% # remove zero variance variables  step_nzv(all_predictors(),-all_outcomes())%>% # remove near zero variance variables  step_normalize(all_numeric(),-all_outcomes())%>% # normalize all numeric features  step_dummy(all_nominal(),-all_outcomes()) %>% # one hot encoding for factor variables  step_corr(all_predictors(),-all_outcomes(), threshold = 0.9) # remove variables that have large absolute correlations with other variables. The step will try to remove the minimum number of columns so that all the resulting absolute correlations are less than this value.  #examine what modifications are done on the dataset  set.seed(my_seed)  prep<-prep(recipe, db_train)  view(juice(prep))  #Examining all steps in the blue print  tidy(prep)  #Examining mean and standard deviation for normalizing AGE  tidy(prep, number= 6)  #examine the development dataset after prep-rocessing  prep[["template"]]  #### (5) Algorithm training ####  ### define performance metrics and training parameters ####  ## define performance metrics  MySummary <- function(data, lev = NULL, model = NULL){  a1 <- defaultSummary(data, lev, model)  b1 <- twoClassSummary(data, lev, model)  c1 <- prSummary(data, lev, model)  out <- c(a1, b1, c1)  out}  ## define training parameters  cv <- trainControl(  method = "repeatedcv",  number = 10,  repeats = 3,  search = "grid",  verboseIter= TRUE,  classProbs = TRUE,  returnResamp = "final",  savePredictions = "final",  summaryFunction = MySummary,  selectionFunction = "tolerance",  allowParallel=TRUE  )  adaptControl <- trainControl(method = "adaptive_cv",  number = 10, repeats = 3,  adaptive = list(min = 5, alpha = 0.05,  method = "gls", complete = TRUE),  search = "random",  verboseIter= TRUE,  classProbs = TRUE,  returnResamp = "final",  savePredictions = "final",  summaryFunction = MySummary,  selectionFunction = "tolerance",  allowParallel=TRUE  )  ### GLM - elastic net ####  hyper_grid_glm <- expand.grid(  alpha = seq(from=0.01, to= 1, by=0.01),  lambda = seq(from=0.01, to= 1, by=0.01)  )  library(doParallel)  no_cores<- detectCores()-2  cl<-makePSOCKcluster(no_cores)  registerDoParallel(cl)  set.seed(my_seed)  cv_glm <- caret::train(recipe,  data=db_train,  method="glmnet",  metric="Kappa",  #tuneLength = 30, #allows system to tune algorithm automatically. It indicates the number of different values to try for each tunning parameter. For example, mtry for randomForest. Suppose, tuneLength = 5, it means try 5 different mtry values and find the optimal mtry value based on these 5 values  trControl=cv,  tuneGrid = hyper_grid_glm  )  stopCluster(cl)  registerDoSEQ()  cv_glm$bestTune  cv_glm$results[c(11),]  ggplot(cv_glm)  set.seed(my_seed)  cv_glm_adapt <- caret::train(recipe,  data=db_train,  method="glmnet",  metric="Kappa",  tuneLength = 30, #allows system to tune algorithm automatically. It indicates the number of different values to try for each tunning parameter. For example, mtry for randomForest. Suppose, tuneLength = 5, it means try 5 different mtry values and find the optimal mtry value based on these 5 values  trControl=adaptControl  #tuneGrid = hyper_grid_glm  )  cv_glm_adapt$bestTune  cv_glm_adapt$results[c(25),]  ggplot(cv_glm)  ### XGBoost ####  hyper_grid_xgboost <- expand.grid(  nrounds = seq(from=25, to= 100, by=25),  max_depth = seq(from=5, to= 35, by=10),  eta = seq(from=0.2, to= 1, by=0.2),  gamma = seq(from=1, to= 10, by=1),  colsample_bytree = seq(from=0.6, to= 1, by=0.2),  min_child_weight = seq(from=2, to= 5, by=1),  subsample = 1  )  library(doParallel)  no_cores<- detectCores()-2  cl<-makePSOCKcluster(no_cores)  registerDoParallel(cl)  set.seed(my_seed)  cv_xgboost <- caret::train(recipe,  data=db_train,  method="xgbTree",  metric="Kappa",  #tuneLength = 30, #allows system to tune algorithm automatically. It indicates the number of different values to try for each tunning parameter. For example, mtry for randomForest. Suppose, tuneLength = 5, it means try 5 different mtry values and find the optimal mtry value based on these 5 values  trControl=cv,  tuneGrid = hyper_grid_xgboost  )  stopCluster(cl)  registerDoSEQ()  cv_xgboost$bestTune  cv_xgboost$results[c("93"),]  ### MARS - Multivariate Adaptive Regression Spline ####  hyper_grid_mars <- expand.grid(  degree = seq(from=1, to= 3, by=1), #no decimals  nprune = seq(from=1, to= 10, by=1) #1  )  library(doParallel)  no_cores<- detectCores()-2  cl<-makePSOCKcluster(no_cores)  registerDoParallel(cl)  set.seed(my_seed)  cv_mars <- caret::train(recipe,  data=db_train,  method="earth",  metric="Kappa",  #tuneLength = 30, #allows system to tune algorithm automatically. It indicates the number of different values to try for each tunning parameter. For example, mtry for randomForest. Suppose, tuneLength = 5, it means try 5 different mtry values and find the optimal mtry value based on these 5 values  trControl=cv,  tuneGrid = hyper_grid_mars  )  stopCluster(cl)  registerDoSEQ()  cv_mars$bestTune  cv_mars$results[c("4"),]  ### SVM - Support Vector Machine ####  #use random search to make it quicker  cv_svm <- trainControl(  method = "repeatedcv",  number = 10,  repeats = 1,  search = "random" ,  verboseIter= TRUE,  classProbs = TRUE,  returnResamp = "final",  savePredictions = "final",  summaryFunction = MySummary,  selectionFunction = "tolerance",  allowParallel=TRUE  )  hyper_grid_svm <- expand.grid(  degree = seq(from=1, to= 11, by=2),  scale = seq(from=0.1, to= 1, by=0.1),  C = seq(from=0.5, to= 8, by=0.5)  )  library(doParallel)  no_cores<- detectCores()-2  cl<-makePSOCKcluster(no_cores)  registerDoParallel(cl)  set.seed(my_seed)  cv_svm <- caret::train(recipe,  data=db_train,  method="svmPoly",  metric="Kappa",  tuneLength = 30, #allows system to tune algorithm automatically. It indicates the number of different values to try for each tunning parameter. For example, mtry for randomForest. Suppose, tuneLength = 5, it means try 5 different mtry values and find the optimal mtry value based on these 5 values  trControl=cv_svm  )  stopCluster(cl)  registerDoSEQ()  plot(cv_svm$history, xlim=c(0,100), ylim=c(0,1))  cv_svm$bestTune  cv_svm$results[c("9"),]  ### Neural network ####  cv_nn <- trainControl(  method = "repeatedcv",  number = 10,  repeats = 1,  search = "random" ,  verboseIter= TRUE,  classProbs = TRUE,  returnResamp = "final",  savePredictions = "final",  summaryFunction = MySummary,  selectionFunction = "tolerance",  allowParallel=TRUE  )  hyper_grid_nn <- expand.grid(  size = seq(from=1, to= 21, by=10),  dropout = seq(from=0.1, to= 0.3, by=0.1),  batch_size = seq(from=1, to= 11, by=5),  lr = seq(from=0.25, to= 1, by=0.25),  rho = seq(from=0.25, to= 1, by=0.25),  decay = seq(from=0.1, to= 0.5, by=0.2),  cost = seq(from=0.25, to= 1, by=0.25),  activation = 'relu'  )  library(doParallel)  no_cores<- detectCores()-2  cl<-makePSOCKcluster(no_cores)  registerDoParallel(cl)  set.seed(my_seed)  cv_nn <- caret::train(recipe,  data=db_train,  method="mlpKerasDropoutCost",  metric="Kappa",  tuneLength = 30, #allows system to tune algorithm automatically. It indicates the number of different values to try for each tunning parameter. For example, mtry for randomForest. Suppose, tuneLength = 5, it means try 5 different mtry values and find the optimal mtry value based on these 5 values  trControl=cv_nn  )  stopCluster(cl)  registerDoSEQ()  plot(cv_nn$history)  cv_nn$bestTune  cv_nn$results[28,]  #### (6) Resampling performance testing ####  ### metrics ####  #glm - elastic net  cv_glm$bestTune  cv_glm$results[c(11),]  cv_glm[["resample"]][["ROC"]] #AUROC  range(cv_glm[["resample"]][["ROC"]])  mean((cv_glm[["resample"]][["ROC"]]))  CI(cv_glm[["resample"]][["ROC"]], ci=0.95)  sd(cv_glm[["resample"]][["ROC"]])  cv_glm[["resample"]][["Kappa"]]  CI(cv_glm[["resample"]][["Kappa"]], ci=0.95)  cv_glm[["resample"]][["Accuracy"]]  CI(cv_glm[["resample"]][["Accuracy"]], ci=0.95)  cv_glm[["resample"]][["Sens"]]  CI(cv_glm[["resample"]][["Sens"]], ci=0.95)  cv_glm[["resample"]][["Spec"]]  CI(cv_glm[["resample"]][["Spec"]], ci=0.95)  cv_glm[["resample"]][["Precision"]] #positive-predictive value  CI(cv_glm[["resample"]][["Precision"]], ci=0.95)  #xgboost  cv_xgboost[["resample"]][["ROC"]] #AUROC  CI(cv_xgboost[["resample"]][["ROC"]], ci=0.95)  range(cv_xgboost[["resample"]][["ROC"]])  cv_xgboost[["resample"]][["Kappa"]]  CI(cv_xgboost[["resample"]][["Kappa"]], ci=0.95)  cv_xgboost[["resample"]][["Accuracy"]]  CI(cv_xgboost[["resample"]][["Accuracy"]], ci=0.95)  cv_xgboost[["resample"]][["Sens"]]  CI(cv_xgboost[["resample"]][["Sens"]], ci=0.95)  cv_xgboost[["resample"]][["Spec"]]  CI(cv_xgboost[["resample"]][["Spec"]], ci=0.95)  cv_xgboost[["resample"]][["Precision"]] #positive-predictive value  CI(cv_xgboost[["resample"]][["Precision"]], ci=0.95)  #mars  cv_mars[["resample"]][["ROC"]] #AUROC  CI(cv_mars[["resample"]][["ROC"]], ci=0.95)  range(cv_mars[["resample"]][["ROC"]])  cv_mars[["resample"]][["Kappa"]]  CI(cv_mars[["resample"]][["Kappa"]], ci=0.95)  cv_mars[["resample"]][["Accuracy"]]  CI(cv_mars[["resample"]][["Accuracy"]], ci=0.95)  cv_mars[["resample"]][["Sens"]]  CI(cv_mars[["resample"]][["Sens"]], ci=0.95)  cv_mars[["resample"]][["Spec"]]  CI(cv_mars[["resample"]][["Spec"]], ci=0.95)  cv_mars[["resample"]][["Precision"]] #positive-predictive value  CI(cv_mars[["resample"]][["Precision"]], ci=0.95)  #svm  cv_svm[["resample"]][["ROC"]] #AUROC  CI(cv_svm[["resample"]][["ROC"]], ci=0.95)  range(cv_svm[["resample"]][["ROC"]])  cv_svm[["resample"]][["Kappa"]]  CI(cv_svm[["resample"]][["Kappa"]], ci=0.95)  cv_svm[["resample"]][["Accuracy"]]  CI(cv_svm[["resample"]][["Accuracy"]], ci=0.95)  cv_svm[["resample"]][["Sens"]]  CI(cv_svm[["resample"]][["Sens"]], ci=0.95)  cv_svm[["resample"]][["Spec"]]  CI(cv_svm[["resample"]][["Spec"]], ci=0.95)  cv_svm[["resample"]][["Precision"]] #positive-predictive value  CI(cv_svm[["resample"]][["Precision"]], ci=0.95)  #nn  cv_nn[["resample"]][["ROC"]] #AUROC  CI(cv_nn[["resample"]][["ROC"]], ci=0.95)  range(cv_nn[["resample"]][["ROC"]])  cv_nn[["resample"]][["Kappa"]]  CI(cv_nn[["resample"]][["Kappa"]], ci=0.95)  cv_nn[["resample"]][["Accuracy"]]  CI(cv_nn[["resample"]][["Accuracy"]], ci=0.95)  cv_nn[["resample"]][["Sens"]]  CI(cv_nn[["resample"]][["Sens"]], ci=0.95)  cv_nn[["resample"]][["Spec"]]  CI(cv_nn[["resample"]][["Spec"]], ci=0.95)  cv_nn[["resample"]][["Precision"]] #positive-predictive value  CI(cv_nn[["resample"]][["Precision"]], ci=0.95)  ### confusion matrices ####  ##glm - elastic net  confusionMatrix(as.factor(cv_glm[["pred"]][["pred"]]), factor(cv_glm[["pred"]][["obs"]]), positive="Malignant") #confusion matrix  ##xgboost  confusionMatrix(as.factor(cv_xgboost[["pred"]][["pred"]]), factor(cv_xgboost[["pred"]][["obs"]]), positive="Malignant") #confusion matrix  ##mars  confusionMatrix(as.factor(cv_mars[["pred"]][["pred"]]), factor(cv_mars[["pred"]][["obs"]]), positive="Malignant") #confusion matrix  ##svm  confusionMatrix(as.factor(cv_svm[["pred"]][["pred"]]), factor(cv_svm[["pred"]][["obs"]]), positive="Malignant") #confusion matrix  ##nn  confusionMatrix(as.factor(cv_nn[["pred"]][["pred"]]), factor(cv_nn[["pred"]][["obs"]]), positive="Malignant") #confusion matrix  #### calibration ####  ## calibration plots  ##glm  glm_calplot_cv <- calibration(factor(cv_glm[["pred"]][["obs"]]) ~ cv_glm[["pred"]][["Malignant"]], data = cv_glm, cuts=10)  xyplot(glm_calplot_cv, auto.key = list(columns = 2),  xlab=list(  label="Predicted Probability",  cex=1.5),  ylab=list(  label="Observed Probability",  cex=1.5),  scales=list(cex=1.5)  )  ggplot(glm_calplot_cv)  ##mars  mars_calplot_cv <- calibration(factor(cv_mars[["pred"]][["obs"]]) ~ cv_mars[["pred"]][["Malignant"]], data = cv_mars, cuts=10)  xyplot(mars_calplot_cv, auto.key = list(columns = 2),  xlab=list(  label="Predicted Probability",  cex=1.5),  ylab=list(  label="Observed Probability",  cex=1.5),  scales=list(cex=1.5)  )  ggplot(mars_calplot_cv)  ##xgboost  xgboost_calplot_cv <- calibration(factor(cv_xgboost[["pred"]][["obs"]]) ~ cv_xgboost[["pred"]][["Malignant"]], data = cv_xgboost, cuts=10)  xyplot(xgboost_calplot_cv, auto.key = list(columns = 2),  xlab=list(  label="Predicted Probability",  cex=1.5),  ylab=list(  label="Observed Probability",  cex=1.5),  scales=list(cex=1.5)  )  ggplot(xgboost_calplot_cv)  ##svm  svm_calplot_cv <- calibration(factor(cv_svm[["pred"]][["obs"]]) ~ cv_svm[["pred"]][["Malignant"]], data = cv_svm, cuts=10)  xyplot(svm_calplot_cv, auto.key = list(columns = 2),  xlab=list(  label="Predicted Probability",  cex=1.5),  ylab=list(  label="Observed Probability",  cex=1.5),  scales=list(cex=1.5)  )  ggplot(svm_calplot_cv)  ##nn  nn_calplot_cv <- calibration(factor(cv_nn[["pred"]][["obs"]]) ~ cv_nn[["pred"]][["Malignant"]], data = cv_nn, cuts=10)  xyplot(nn_calplot_cv, auto.key = list(columns = 2),  xlab=list(  label="Predicted Probability",  cex=1.5),  ylab=list(  label="Observed Probability",  cex=1.5),  scales=list(cex=1.5)  )  ggplot(nn_calplot_cv)  ## calibration scores  Spiegelhalter_z = function(y, prob){  alpha = 0.05  z_score = sum((y-prob)*(1-2*prob))/sqrt(sum(((1-2*prob)^2)*prob*(1-prob)))  print(z_score)  if (abs(z_score) > qnorm(1-alpha/2)){  print('reject null. NOT calibrated')  } else{  print('fail to reject. calibrated')  }  cat('z score: ', z_score, '\n')  cat('p value: ', 1-pnorm(abs(z_score)), '\n')  return(z_score)  }  ##glm elastic net  Spiegelhalter_z (unfactor(revalue(cv_glm[["pred"]][["obs"]], c("Malignant"=1, "Benign"=0))), cv_glm[["pred"]][["Malignant"]])  ##xgboost  Spiegelhalter_z (unfactor(revalue(cv_xgboost[["pred"]][["obs"]], c("Malignant"=1, "Benign"=0))), cv_xgboost[["pred"]][["Malignant"]])  ##mars  Spiegelhalter_z (unfactor(revalue(cv_mars[["pred"]][["obs"]], c("Malignant"=1, "Benign"=0))), cv_mars[["pred"]][["Malignant"]])  ##svm  Spiegelhalter_z (unfactor(revalue(cv_svm[["pred"]][["obs"]], c("Malignant"=1, "Benign"=0))), cv_svm[["pred"]][["Malignant"]])  ##nn  Spiegelhalter_z (unfactor(revalue(cv_nn[["pred"]][["obs"]], c("Malignant"=1, "Benign"=0))), cv_nn[["pred"]][["Malignant"]])  #### (7) (External) validation ####  #predict probabilities  predict(cv_glm , db_test, type = "prob")  #predict classes  predict(cv_glm , db_test)  #apply pre-processing steps to validation set manually (done automatically by "predict")  bake(prep, db_test)  ### ROC ####  roc_glm_validation = roc(as.vector(db_test$outcome),as.matrix(predict(cv_glm , db_test, type = "prob")$"Malignant")) #Conduct the ROC analyses  auc_glm_validation = pROC::auc(roc_glm_validation) #Calculate the area under the ROC curve  auc_CI_glm_validation = pROC::ci.auc(roc_glm_validation, method="bootstrap", boot.stratified=TRUE) #Calculate the area under the ROC curve  roc_glm_adapt_validation = roc(as.vector(db_test$outcome),as.matrix(predict(cv_glm_adapt , db_test, type = "prob")$"Malignant")) #Conduct the ROC analyses  auc_glm_adapt_validation = pROC::auc(roc_glm_adapt_validation) #Calculate the area under the ROC curve  auc_CI_glm_adapt_validation = pROC::ci.auc(roc_glm_adapt_validation, method="bootstrap", boot.stratified=TRUE) #Calculate the area under the ROC curve  roc_xgboost_validation = roc(as.vector(db_test$outcome),as.matrix(predict(cv_xgboost , db_test, type = "prob")$"Malignant")) #Conduct the ROC analyses  auc_xgboost_validation = pROC::auc(roc_xgboost_validation) #Calculate the area under the ROC curve  auc_CI_xgboost_validation = pROC::ci.auc(roc_xgboost_validation, method="bootstrap", boot.stratified=TRUE) #Calculate the area under the ROC curve  roc_mars_validation = roc(as.vector(db_test$outcome),as.matrix(predict(cv_mars , db_test, type = "prob")$"Malignant")) #Conduct the ROC analyses  auc_mars_validation = pROC::auc(roc_mars_validation) #Calculate the area under the ROC curve  auc_CI_mars_validation = pROC::ci.auc(roc_mars_validation, method="bootstrap", boot.stratified=TRUE) #Calculate the area under the ROC curve  roc_svm_validation = roc(as.vector(db_test$outcome),as.matrix(predict(cv_svm , db_test, type = "prob")$"Malignant")) #Conduct the ROC analyses  auc_svm_validation = pROC::auc(roc_svm_validation) #Calculate the area under the ROC curve  auc_CI_svm_validation = pROC::ci.auc(roc_svm_validation, method="bootstrap", boot.stratified=TRUE) #Calculate the area under the ROC curve  roc_nn_validation = roc(as.vector(db_test$outcome),as.matrix(predict(cv_nn , db_test, type = "prob")$"Malignant")) #Conduct the ROC analyses  auc_nn_validation = pROC::auc(roc_nn_validation) #Calculate the area under the ROC curve  auc_CI_nn_validation = pROC::ci.auc(roc_nn_validation, method="bootstrap", boot.stratified=TRUE) #Calculate the area under the ROC curve  plot.roc(roc_glm_validation, ylim=c(0,1), xlim=c(1,0), cex.lab=1.8, cex.axis=1.5, cex.main=1.8, cex.sub=1.8,  legacy.axes=TRUE) #Plot the ROC curves  lines(roc_glm_validation, col="blue")  lines(roc_xgboost_validation, col="red")  lines(roc_mars_validation, col="orange")  lines(roc_svm_validation, col="black")  lines(roc_nn_validation, col="grey60")  legend("bottomright", legend=c("LR with Elastic Net Penalty", "XGBoost Tree", "MARS", "SVM", "neural network"), col=c("blue", "red","orange", "black", "grey60"), lwd=2, cex=1.3)  #### confusion matrices ####  #glm - elastic net  confusionMatrix(as.factor(predict(cv_glm , db_test)), factor(db_test$outcome), positive="Malignant") #confusion matrix  #xgboost  confusionMatrix(as.factor(predict(cv_xgboost , db_test)), factor(db_test$outcome), positive="Malignant") #confusion matrix  #mars  confusionMatrix(as.factor(predict(cv_mars , db_test)), factor(db_test$outcome), positive="Malignant") #confusion matrix  #svm  confusionMatrix(as.factor(predict(cv_svm , db_test)), factor(db_test$outcome), positive="Malignant") #confusion matrix  #nn  confusionMatrix(as.factor(predict(cv_nn , db_test)), factor(db_test$outcome), positive="Malignant") #confusion matrix  #### calibration ####  ## calibration plots  ##glm  glm_calplot_validation <- calibration(factor(db_test$outcome) ~ as.matrix(predict(cv_glm , db_test, type = "prob")$"Malignant"), data = db_test, cuts=10)  xyplot(glm_calplot_validation, auto.key = list(columns = 2),  xlab=list(  label="Predicted Probability",  cex=1.5),  ylab=list(  label="Observed Probability",  cex=1.5),  scales=list(cex=1.5)  )  ggplot(glm_calplot_validation)  ##mars  mars_calplot_validation <- calibration(factor(db_test$outcome) ~  as.matrix(predict(cv_mars , db_test, type = "prob")$"Malignant"),  data = db_test, cuts=10)  xyplot(mars_calplot_validation, auto.key = list(columns = 2),  xlab=list(  label="Predicted Probability",  cex=1.5),  ylab=list(  label="Observed Probability",  cex=1.5),  scales=list(cex=1.5)  )  ggplot(mars_calplot_validation)  ##xgboost  xgboost_calplot_validation <- calibration(factor(db_test$outcome) ~ as.matrix(predict(cv_xgboost , db_test, type = "prob")$"Malignant"), data = db_test, cuts=10)  xyplot(xgboost_calplot_validation, auto.key = list(columns = 2),  xlab=list(  label="Predicted Probability",  cex=1.5),  ylab=list(  label="Observed Probability",  cex=1.5),  scales=list(cex=1.5)  )  ggplot(xgboost_calplot_validation)  ##svm  svm_calplot_validation <- calibration(factor(db_test$outcome) ~ as.matrix(predict(cv_svm , db_test, type = "prob")$"Malignant"), data = db_test, cuts=10)  xyplot(svm_calplot_validation, auto.key = list(columns = 2),  xlab=list(  label="Predicted Probability",  cex=1.5),  ylab=list(  label="Observed Probability",  cex=1.5),  scales=list(cex=1.5)  )  ggplot(svm_calplot_validation)  ##nn  nn_calplot_validation <- calibration(factor(db_test$outcome) ~ as.matrix(predict(cv_nn , db_test, type = "prob")$"Malignant"), data = db_test, cuts=10)  xyplot(nn_calplot_validation, auto.key = list(columns = 2),  xlab=list(  label="Predicted Probability",  cex=1.5),  ylab=list(  label="Observed Probability",  cex=1.5),  scales=list(cex=1.5)  )  ggplot(nn_calplot_validation)  ## calibration scores  ##glm elastic net  Spiegelhalter_z (unfactor(revalue(db_test$outcome, c("Malignant"=1, "Benign"=0))), as.matrix(predict(cv_glm , db_test, type = "prob")$"Malignant"))  ##xgboost  Spiegelhalter_z (unfactor(revalue(db_test$outcome, c("Malignant"=1, "Benign"=0))), as.matrix(predict(cv_xgboost , db_test, type = "prob")$"Malignant"))  ##mars  Spiegelhalter_z (unfactor(revalue(db_test$outcome, c("Malignant"=1, "Benign"=0))), as.matrix(predict(cv_mars , db_test, type = "prob")$"Malignant"))  ##svm  Spiegelhalter_z (unfactor(revalue(db_test$outcome, c("Malignant"=1, "Benign"=0))), as.matrix(predict(cv_svm , db_test, type = "prob")$"Malignant"))  ##nn  Spiegelhalter_z (unfactor(revalue(db_test$outcome, c("Malignant"=1, "Benign"=0))), as.matrix(predict(cv_nn , db_test, type = "prob")$"Malignant"))  #### (8) compare Performance ####  ###AUC ####  # ML vs. ML  roc.test(roc_glm_validation, roc_xgboost_validation, method="bootstrap", alternative = "two.sided", boot.n=2000, boot.stratified=TRUE)  roc.test(roc_glm_validation, roc_mars_validation, method="bootstrap", alternative = "two.sided", boot.n=2000, boot.stratified=TRUE)  roc.test(roc_glm_validation, roc_svm_validation, method="bootstrap", alternative = "two.sided", boot.n=2000, boot.stratified=TRUE)  roc.test(roc_glm_validation, roc_nn_validation, method="bootstrap", alternative = "two.sided", boot.n=2000, boot.stratified=TRUE)  roc.test(roc_xgboost_validation, roc_mars_validation, method="bootstrap", alternative = "two.sided", boot.n=2000, boot.stratified=TRUE)  roc.test(roc_xgboost_validation, roc_svm_validation, method="bootstrap", alternative = "two.sided", boot.n=2000, boot.stratified=TRUE)  roc.test(roc_xgboost_validation, roc_nn_validation, method="bootstrap", alternative = "two.sided", boot.n=2000, boot.stratified=TRUE)  roc.test(roc_mars_validation, roc_svm_validation, method="bootstrap", alternative = "two.sided", boot.n=2000, boot.stratified=TRUE)  roc.test(roc_mars_validation, roc_nn_validation, method="bootstrap", alternative = "two.sided", boot.n=2000, boot.stratified=TRUE)  roc.test(roc_svm_validation, roc_nn_validation, method="bootstrap", alternative = "two.sided", boot.n=2000, boot.stratified=TRUE)  ### Sensitivity, Specificity, NPV, PPV ####  ##use mcnemar test to compare different models  mcnemar.test( predict(cv_glm , db_test),  predict(cv_mars , db_test),  correct = TRUE)  mcnemar.test( predict(cv_glm , db_test),  predict(cv_xgboost , db_test),  correct = TRUE)  mcnemar.test( predict(cv_glm , db_test),  predict(cv_svm , db_test),  correct = TRUE)  mcnemar.test( predict(cv_glm , db_test),  predict(cv_nn , db_test),  correct = TRUE)  mcnemar.test( predict(cv_mars , db_test),  predict(cv_xgboost , db_test),  correct = TRUE)  mcnemar.test( predict(cv_mars , db_test),  predict(cv_svm , db_test),  correct = TRUE)  mcnemar.test( predict(cv_mars , db_test),  predict(cv_nn , db_test),  correct = TRUE)  mcnemar.test( predict(cv_xgboost , db_test),  predict(cv_svm, db_test),  correct = TRUE)  mcnemar.test( predict(cv_xgboost , db_test),  predict(cv_nn, db_test),  correct = TRUE)  mcnemar.test( predict(cv_svm , db_test),  predict(cv_nn, db_test),  correct = TRUE) |
| --- |
